# Lenalidomide, Melphalan, and Prednisone Association Is an Effective Salvage Therapy in Relapsed Plasma Cell Leukaemia

**DOI:** 10.1155/2009/867380

**Published:** 2009-11-17

**Authors:** Tommasina Guglielmelli, Roberta Merlini, Emilia Giugliano, Giuseppe Saglio

**Affiliations:** Department of Clinical and Biological Sciences, San Luigi Hospital, Regione Gonzole 10, Orbassano 10043, Turin, Italy

## Abstract

Plasma cell leukemia (PCL) is a rare and aggressive plasma cell disorder, characterized by the presence of a peripheral blood absolute plasma cell count of at least 2 × 109/l and more than 20% circulating plasma cells. The prognosis of PCL patients remains poor. Even by using autologous or allogenic transplant procedures, median survival does not exceed 3 years (Saccaro et al., 2005). Thalidomide, bortezomib and lenalidomide (Revlimid) have emerged as high active agents in the treatment of PCL (Johnston and abdalla, 2002; Musto et al., 2007; Finnegan et al., 2006). In particular, Lenalidomide is a structural analogue of thalidomide with similar but more potent biological activity; it is used as first line therapy in MM (Palumbo et al., 2007; Niesvizky et al., 2007), although information regarding its associated use with dexamethasone use as salvage therapy in PCL derives from anecdotal single case reports (Musto et al., 2008). We would like to describe a case of primary PCL with adverse cytogenetic in which excellent response was achieved with the combination of lenalidomide, melphalan, and prednisone as salvage therapy.

## 1. Case Report

A 50-years-old man was diagnosed with PCL in March 2006. He was admitted due to fatigue. The full blood cell count revealed elevated white blood cell count (25 × 10^9^ L^−1^) associated with the presence of 50% peripheral blood plasma cells. Serum immunofixation showed an IgGk monoclonal component (MC) with high IgG immunoglobulin level (10.5 g/dL). 

 Immunophenotyping demonstrated a plasma cells population CD38, CD138,CD40, CD45 , CD20 positive and CD56, CD19 negative. The results of the cytogenetic analysis performed with interphase Fluorescence In Situ Hybridization analysis showed 13q14 deletion, t(4;14) translocation, and p53 deletion. The patient's ISS score was 2 and, importantly, he was diagnosed to be positive for Hepatitis B virus (HBV) surface antigen with normal liver function. HBeAg, HBeAb, HBCAg, and HBV Dna at diagnosis were negative. The management decision was to perform autologous stem cell transplantation (ASCT) following adequate stem cell collection with cyclophosphamide and two cycles of combination chemotherapy with vincristine, doxorubicin, and dexamethasone (VAD). The patient did not respond to the induction therapy and, therefore, he underwent two cycles of combination chemotherapy consisting of bortezomib (1.3 mg/m^2^ d 1, 4, 8, 11), doxorubicin (20 mg d 1.4 ) and dexamethasone (40 mg d -2-1, 2, 3, 4). A partial response (PR) was documented (MC component reduction 85%) and the patients underwent autologous stem cell transplantation with melphalan 200 mg/m^2^ as conditioning regimen. He achieved a very good partial response [1]. A reactivation of HBV infection leading to an acute hepatitis (HBV DNA >106 copies/mL) was diagnosed two months after the transplant despite antiviral therapy with lamivudine and the patient was treated with combination antiviral therapy with lamivudine and adefovir dipivoxil for six months until hepatitis resolution. A tandem ASCT was excluded for the high risk of hepatic complications. Maintenance therapy with thalidomide 100 mg/d was then started and carried on for 6 months until progressive disease [2]. On May 2008 the patient relapsed with a progressive increase in MC amount (4.3 g/dL), high WBC count (11 × 10^9^ L^−1^) associated with the presence of 30% peripheral blood plasma cells, and advanced bone disease. Due to the relevant side effects observed after the first ASCT, the patient was considered noneligible for allogenic transplantation even if there was an available HLA matched donor. On May 2008 the patient started lenalidomide 25 mg/d for 21 d plus dexamethasone 40 mg d 1–4 for a 28-d cycle for two cycles. As a stable disease was obtained and due to the persistence of bone pain, low dose melphalan (0.1 mg/kg/d d 1–4) was added on the third cycle in order to achieve a rapid response for six additional melphalan-prednisone-lenalidomide (MPR) cycles. Each MPR cycle was administered every 30–35 days and at haematological reconstitution. 

Transient grade 3-4 thrombocytopenia treated with platelets transfusion and transient grade 2-3 neutropenia treated with G-CSF were observed. We do not observe thromboembolic or neurological events during MPR treatment. The patient achieved a PR after 3 MPR cycles (MC reduction 55%, absence of circulating plasma cells) and he remained in PR for four months. On February 2009 the patient relapsed and he underwent autologous transplantation as salvage therapy. He died on April 2009 for progressive disease. 

## 2. Discussion

Plasma cell leukaemia is a rare and aggressive form of multiple myeloma characterized by very poor prognosis. The median overall survival with conventional therapy is about 7–14 months, while even after stem cell transplantation the outcome remains disappointing [3]. Recently, it has been demonstrated that bortezomib may be also effective and safe in the treatment of PCL [4, 5]. A single case report evidenced the effectiveness of the association of prednisone and lenalidomide in relapsed PCL [6]. In a phase 2 study, the association of melphalan-prednisone-lenalidomide (Revlimid) (MPR) was very promising in multiple myeloma patients with 81% of patients achieved at least a partial response, 47.6% achieved a very good partial response, and 23.8% achieved a complete immunofixation-negative response. The EFS and OS at 1 year were 92% and 100%, respectively. A large phase 3 international trial comparing melphalan-prednisone with MPR is currently ongoing. Recent literature suggests the effectiveness of a combination of melphalan-prednisone-lenalidomide (Revlimid) (MPR) in plasma cell disorders, resulting in a highly effective regimen for patients noneligible for high dose therapy and stem cells transplantation [7, 8]. 

To our knowledge, this is the first reported patient with primary PCL treated with standard dose lenalidomide (25 mg/d) plus low dose melphalan (0.1 mg/kg/d d 1–4). No severe adverse event was observed. Moreover, in our patient, MPR regimen has demonstrated enhanced activity despite high risk prognostic factors. We conclude that MPR is safe and effective and could be used in plasma cell leukaemia patients in relapse and/or noneligible for autologous or allogenic bone marrow transplantation. Moreover, MPR regimen may be also considered in PCL as first line therapy or as new induction regimen in the ASCT setting. 
